# 10-year time course of Hg and organic compounds in Augusta Bay: Bioavailability and biological effects in marine organisms

**DOI:** 10.3389/fpubh.2022.968296

**Published:** 2022-09-21

**Authors:** Maura Benedetti, Elena Romano, Antonella Ausili, Daniele Fattorini, Stefania Gorbi, Chiara Maggi, Andrea Salmeri, Daniela Salvagio Manta, Giulio Sesta, Mario Sprovieri, Francesco Regoli

**Affiliations:** ^1^Department of Life and Environmental Sciences, Polytechnic University of Marche, Ancona, Italy; ^2^CoNISMa, Consorzio Interuniversitario per le Scienze del Mare, Rome, Italy; ^3^ISPRA, Italian Institute for Environmental Protection and Research, Rome, Italy; ^4^Institute of Anthropic Impacts and Sustainability in Marine Environment, National Research Council, Trapani, Italy

**Keywords:** mercury, hexachlorobenzene, marine organisms, trophic transfer, biomarkers, bioavailability

## Abstract

In the last century, many Mediterranean coastal areas have been subjected to anthropogenic disturbances from industrial activities, uncontrolled landfills, shipyards, and high maritime traffic. The Augusta Bay (eastern Sicily, Italy) represents an example of a strongly impacted coastal environment with an elevated level of sediments contamination due to the presence of one of the largest European petrochemical plants, combined with an extensive commercial and military harbor. The most significant contaminants were represented by mercury (Hg) and hexachlorobenzene (HCB), derived from a former chlor-alkali plant, and other organic compounds like polycyclic aromatic hydrocarbons (PAHs) and polychlorobiphenyls (PCBs). Since the 1970s, Augusta Bay has become internationally recognized as a contaminated marine environment, although very little information is available regarding the temporal trend of contaminants bioavailability and biological impacts on aquatic organisms. In this study, the Hg and HCB concentrations were investigated over 10 years (from 2003 to 2013) in sediments and invertebrate and vertebrate organisms; these two contaminants' ecotoxicity was further evaluated at a biochemical and cellular level by analyzing the induction of organic biotransformation processes and DNA damages. The results showed high concentrations of Hg and HCB in sediments and their strong bioaccumulation in different species with significantly higher values than those measured in reference sites. This trend was paralleled by increased micronuclei frequency (DNA damage biomarker) and activity of the biotransformation system. While levels of chemicals in sediments remained elevated during the time course, their bioavailability and biological effects showed a gradual decrease after 2003, when the chlor-alkali plant was closed. Environmental persistence of Hg and HCB availability facilitates their bioaccumulation and affects the health status of marine organisms, with possible implications for environmental risk, pollutants transfer, and human health.

## Introduction

In the last century, several coastal areas have been subjected to strong disturbances and impacts arising from a combination of multiple leakages/discharges of anthropogenic activities and inadequate environmental legislation and monitoring (1, 2). Along the Mediterranean Sea, a large variety of industrial activities (e.g., chemical, petrochemical, thermoelectric, nuclear), uncontrolled landfills, intensive agricultural activities, military arsenals, shipyards, and maritime traffic severely altered the natural conditions of several coastal areas (3). The supply of contaminants to the aquatic environment may occur *via* point and diffuse sources, e.g., direct discharge to the sea, the contribution of runoff from contaminated lands, and atmospheric deposition. In this context, marine sediments play a fundamental role, as they are the final sink and the secondary source of most toxic pollutants (4), with a high potential to be bioaccumulated into the food web *via* benthic-pelagic coupling (5, 6).

Persistent contaminants may remain strongly associated with sediments for a long time after the cessation of active inputs (7), with different chemical forms which influence their subsequent fate and impact; chemicals reversibly adsorbed to sediments can represent a particular threat to biota since they are susceptible to re-release into the water in specific hydrological and/or chemical conditions (8, 9). In this context, sediments can represent a source of diffuse pollution in the water compartment affecting both nektonic and benthic organisms (10). Typical contaminants frequently associated with and potentially released from sediments include metals such as cadmium (Cd), copper (Cu), chromium (Cr), mercury (Hg), lead (Pb), and zinc (Zn), as well as organic pollutants including polycyclic aromatic hydrocarbons (PAHs), polychlorinated biphenyls (PCBs), organochlorine pesticides, dioxins and furans (PCDD/F), and organometallic compounds such as tributyltin (TBT). In particular, Hg and organochlorine compounds can be efficiently transferred to biota and biomagnified along the food web reaching high concentrations in the upper trophic levels, thus representing a risk to human health (11, 12). In this context, useful information on the environmental status and pollution effects can be provided by the study of the native and transplanted organisms such as fish (13) and filter feeders (14) through a multidisciplinary, ecotoxicological approach that integrates the results on pollutants bioavailability and bioaccumulation with the onset of biological effects at molecular, biochemical, cellular levels (15, 16). Such biomarkers are strongly recommended in environmental monitoring programs due to their quick response and predictive value, and their use has already been applied in several contaminated sites, including harbor areas, offshore platforms, and petrochemical plants (16–18).

A wide range of environmental contaminants induces biotransformation responses *via* the aryl hydrocarbon receptor (AhR) through a process catalyzed by the cytochrome P450 (CYP) (19). The superfamily of CYP 1 is modulated by a wide variety of xenobiotic compounds, including hexachlorobenzene (HCB), PCBs, and PAHs; its specific enzymatic activity (EROD) is extensively used in biomonitoring programs as a specific marker of exposure to these organic compounds in vertebrate species (20, 21). The production of reactive oxygen species (ROS) and the organic metabolites due to AhR induction can cause severe consequences on biological macromolecules such as proteins, lipids, and DNA (22). Oxidative DNA damage is often used as an indicator of the effects of chemical pollutants (15), and a wide literature established the positive correlation between contaminants and DNA damage, in particular the micronuclei assay (23, 24), confirming genotoxicity as a useful tool to determine the impact of pollutants in monitoring programs (25, 26).

**Figure 1:**
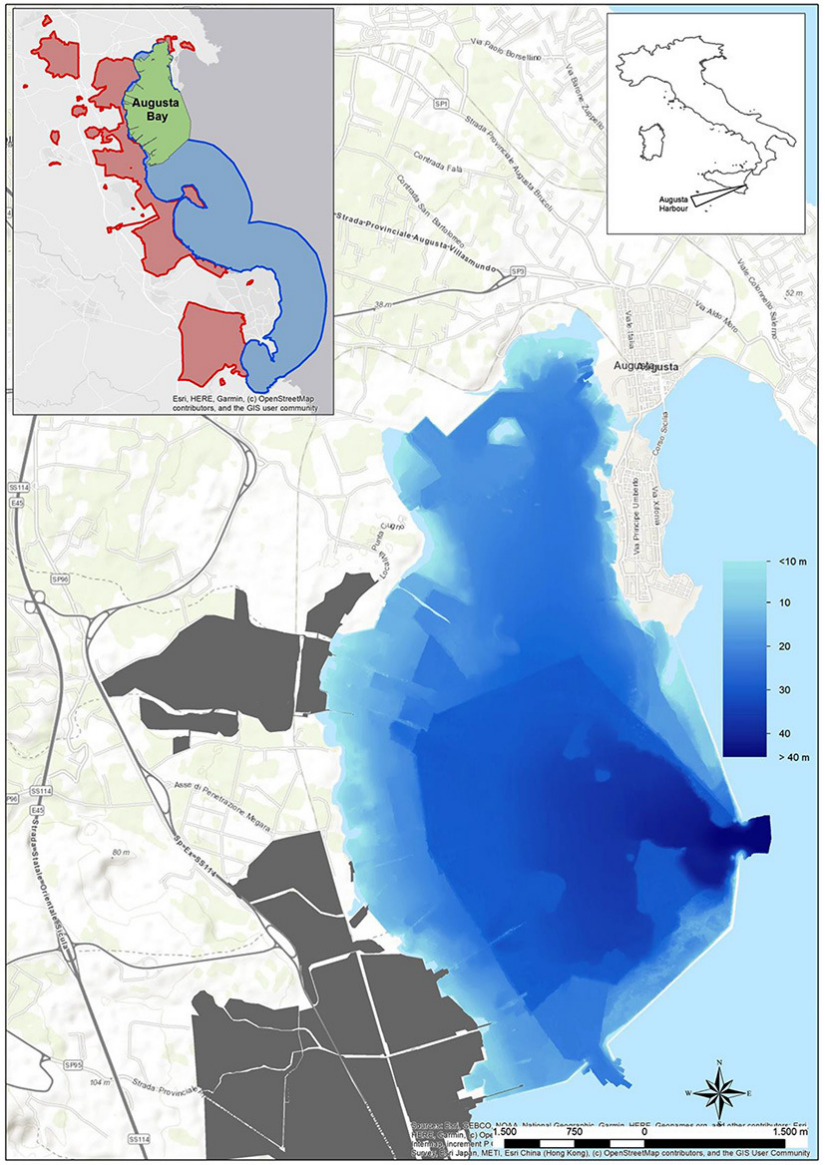
Augusta Bay with the sea bottom depth. In gray color, the industrial area. In the small box is reported, in addition to Augusta Bay (green area), the whole extension of the Site of National Interest named “Priolo-Gargallo” on land (red area) and sea (blue area).

Since the 1970s, the highly industrialized area of Augusta Bay (East Sicily, Italy) has become internationally recognized as a contaminated marine environment. The pollutant of greatest concern in sediments was Hg, derived from a former chlor-alkali plant, accomplished by other metals and organic compounds, such as PAHs, HCB, and PCBs mainly discharged from co-located petrochemical industries. For these reasons, the area was included in the Sites of National Interest (SIN) and named “Priolo-Gargallo” by the Italian Ministry of the Environment in 2003: the site is characterized by high complexity and diversity as regards the natural setting as well as extent, history, type, and degree of contamination. The natural bay of Augusta, containing the harbor and several industrial installations, is 8 km long and 4 km wide, bordered on the southern sector by breakwaters built in the early 1960s. In addition to the harbor and Augusta Bay, the SIN includes a very large marine coastal area up to Siracusa, extending up to 3 km offshore (Figure 1).

Augusta Bay is characterized by intense commercial and industrial maritime activities and a huge chemical and petrochemical pole, operating for several decades. It represents one of the largest European petrochemical poles, which determined the strong pollution of marine sediments. Industrial activities started in the 1950s and quickly developed until the 1980s; afterward, some industries were dismissed, whereas others are still active. In particular, a chlor-alkali plant, based on Hg-cell technology and in operation from 1958 to 2003, was considered of high environmental concern for the marine area owing to the discharge of over 500 tons of Hg directly into the sea in a historical period during which no environmental legislation was active (27).

Several previous investigations established the magnitude of some contaminants in sediments (27–29). The severe Hg, HCB, and PCBs contamination resulted in a marked bioavailability and bioaccumulation of these contaminants in several marine species (17, 30, 31) and, in particular, for Hg contents, the regulatory limits for food consumption in the tissues of fish and mussels were exceeded (2, 11, 15, 17, 28, 32). This critical environmental condition affected marine life, and some biological effects in terms of oxidative and DNA damage were revealed in marine organisms (15, 17).

In this study, an integrated chemical and ecotoxicological approach was applied in a temporal window of 10 years (2003–2013) to obtain information on the degree and temporal trend of marine organisms' contamination due to the main pollutants of the area (Hg and HCB) and the environmental status of Augusta Bay; in addition, some biological alterations and early adverse effects caused by pollutants, in term of DNA damage (micronuclei frequency) and induction of organic biotransformation pathway (CYP450 activity) were evaluated. The micronucleus test was successfully applied in many field studies using bivalves and fish as bioindicator organisms to assess genotoxic compounds, including Hg and organic pollutants, and their effects on the marine environment (33). Concerning the organic biotransformation pathway, the activity of ethoxyresorufin O-deethylase (EROD) appears to be the most sensitive catalytic analysis for determining the induction of the CYP450 system in fish, and it represents a biomarker of specific exposure to organic chemicals like PCB, HCB and PAHs (19).

## Materials and methods

### Sampling activity

**Figure 2:**
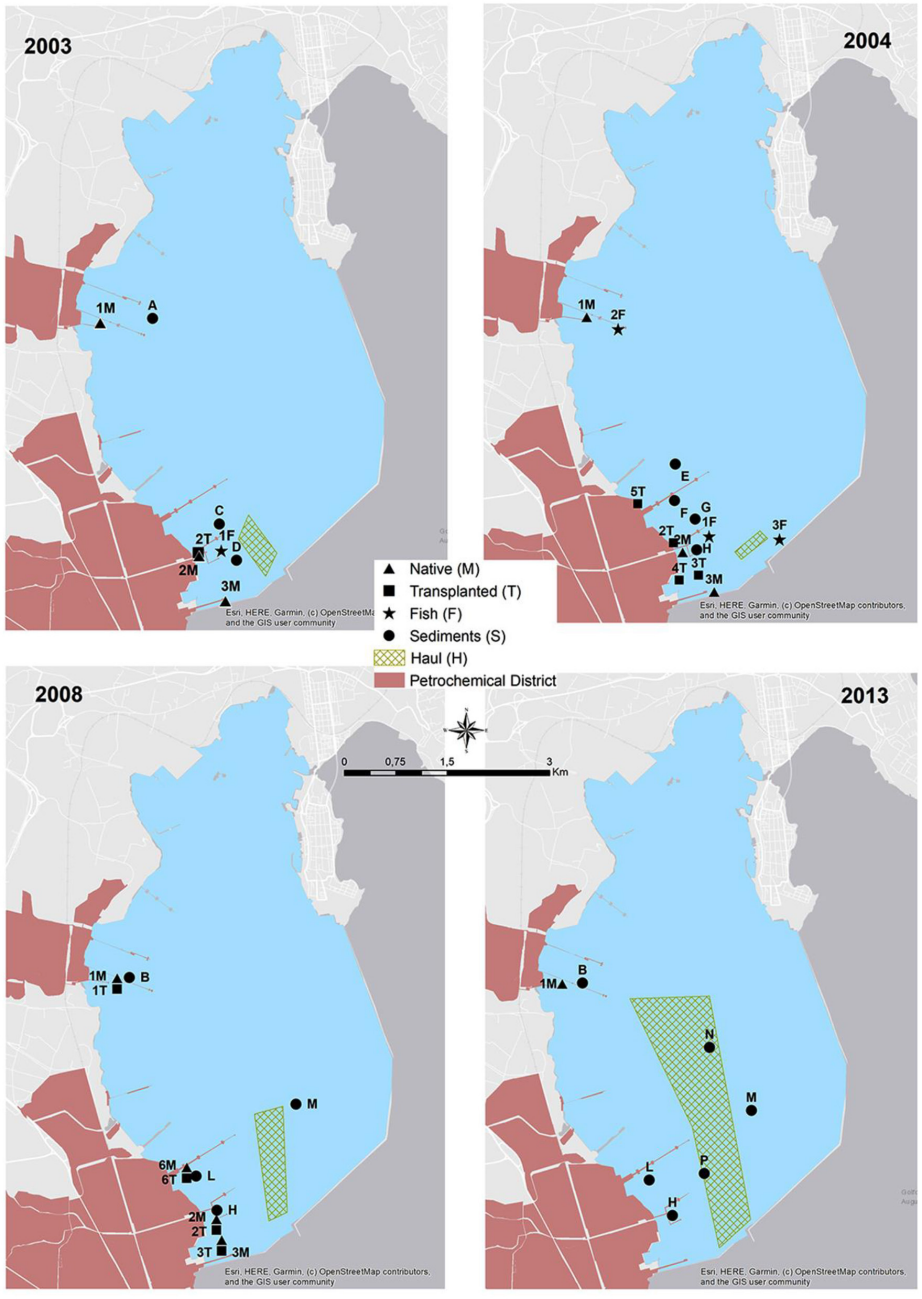
Sediment sampling stations (from A to P), native mussels stations (from 1M to 6M), transplanted mussels stations (from 1T to 6T), fish stations (from 1F to 3F) from 2003 to 2013 and hauls for different years (1H, 2003; 2H, 2004; 3H, 2008; 4H, 2013).

Sediments and marine organisms were collected from different sectors of Augusta Bay in the spring of 2003, 2004, 2008, and December 2013. The sampling strategy considered the presence of industrial settlements with different production characteristics, mainly petroleum-related in the central-western sector of the bay and petrochemical activities in the southern one. The sediments collected are part of more extensive survey campaigns to identify industrial activities' potential impact on the marine area. The sediment cores were collected in three representative areas where native and transplanted mussels were also analyzed (S1, S2, and S3) and in a fourth one (S4) representative of the sector where fish samples were collected (Figure 2; Table 1).

**Table 1:** Sediment cores for each sector. Letters for A to P represent the sampling stations (cfr. Figure 2).

**AREAS/YEAR**	**2003**	**2004**		**2008**	**2013**		
**S1**	A			B	B		
**S2**	C	E	F	L	L		
**S3**	D	G	H	H	H		
**S4**				M	P	N	M

Sediment cores were collected using a gravity corer and subsequently sub-sampled by extrusion at 3 cm intervals for determination of grain size and Hg and HCB contents; each level was identified and separated in different bags and stored at −20°C until the chemical-physical analysis. In this paper, only the top layer (0–15 cm) was considered.

During each sampling year, the collection of Mediterranean mussels, *Mytilus galloprovincialis*, native and caged ones, and different fish species was carried out (Table 2; Figure 2). In detail, the mussels with a size range of 3.6–6.0 cm were manually collected from industrial piers and rapidly transported to the laboratory for tissue dissection. Contextually, mussels of similar size, obtained from a commercial farm far from petrochemical input, were divided into different pools. A pool of individuals was used as control (CTRL) while other pools were transplanted in two piers close to the two main petrochemical districts (Figure 2). The caging was performed using nylon net bags, and the mussels were placed at 3 m water depth for 4 weeks. After collecting native and transplanted mussels, the whole tissues of 25 organisms for every site were dissected and stored at −20°C for chemical analyses. In addition, an aliquot of haemolymph was withdrawn from the adductor muscle of 30 specimens (10 pools) and immediately fixed in Carnoy solution (1:3 acetic acid:methanol) and maintained at +4°C for micronuclei analysis; the only exception was represented by mussels collected in 2003, in which the micronuclei analysis was not carried out. In the same period, different fish specimens were obtained by local fishermen (gill nets-H) and by spearfishing (F), as reported in Figure 2. In detail, 55, 90, 53, and 67 specimens of fish species were collected in 2003, 2004, 2008, and 2013, respectively.

**Table 2:** List and number of collected finfish species in Augusta Bay sampling years.

**Date**	**2003**	**2004**	**2008**	**2013**
**Species**	*Boops salpa* (*n =* 4)	*Boops salpa* (*n =* 4)	*Myliobatis aquila* (*n =* 3)	*Diplodus sp. (n = 10)*
	*Epinephelus aeneus* (*n =* 1)	*Dentex dentex* (*n =* 2)	*Mullus barbatus (n = 25)*	*Mullus barbatus (n = 30)*
	*Merluccius merluccius* (*n =* 2)	*Dicentrarchus labrax* (*n =* 1)	*Pagellus erythrinus* (*n =* 4)	*Pagellus erythrinus (n = 15)*
	*Mullus barbatus (n = 23)*	*Diplodus sp. (n = 33)*	*Serranus cabrilla* (*n =* 6)	*Serranus cabrilla* (*n =* 1)
	*Mullus surmuletus* (*n =* 1)	*Mullus barbatus (n = 26)*	*Serranus hepatus* (*n =* 6)	*Sparus aurata* (*n =* 9)
	*Mugil cephalus* (*n =* 1)	*Mugil cephalus* (*n =* 6)	*Scorpaena notata* (*n =* 6)	*Sphyraena sphyraena* (*n =* 2)
	*Pagellus erythrinus (n = 11)*	*Pagellus erythrinus* (*n =* 4)	*Solea vulgaris* (*n =* 2)	
	*Scorpaena spp*. (*n =* 1)	*Sciaena umbra* (*n =* 2)		
	*Serranus cabrilla* (*n =* 7)	*Scorpaena spp*. (*n =* 6)		
	*Sparus aurata* (*n =* 2)	*Serranus cabrilla* (*n =* 6)		
	*Sphyraena sphyraena* (*n =* 8)			
	*Chelidonichthys lucerna* (*n =* 3)			

After fish collection, livers and muscles were dissected and rapidly frozen in liquid nitrogen for chemical (Hg, HCB) and biochemical analyses, while gills were rapidly fixed in Carnoy solution and maintained at +4°C for micronuclei determination; also for fish, the micronuclei analysis was not carried out in organisms collected in 2003.

### Textural and chemical analysis of marine sediments

Grain-size analyses were conducted according to Romano et al. (34) on pre-treated samples, successively wet-separated into coarse (>63 μm) and fine (<63 μm) fractions. The first was dry-sieved with meshes (ASTM series), ranging from −1 to +4 φ, while the second was analyzed using a laser granulometer (HELOS FKV, Sympatech, Germany). Sediments were then classified according to Shepard (35).

Hg was measured using a Milestone Direct Mercury Analyzer (DMA-80) (EPA Method 7473). The Hg analysis was performed on sediment samples dried at 35°C for 48 h. Quality parameters such as accuracy, quantification limit, and repeatability were estimated to guarantee QA/QC. The accuracy was evaluated using certified reference materials; further details are reported in Supplementary Table 1S.

To determine HCB, the extraction was performed by Pressurized Fluid Extraction (PLE) with Dionex ASE 200 instrument and 60:40 petroleum ether/dichloromethane solvent mixture. A 5 g aliquot was loaded in extraction cells containing a 2.5 g florisil^®^ bed for preliminary purification. Sulfur was removed by elution, and the extract was shaken with concentrated sulphuric acid to complete the purification from organic interferences. The gaschromatographic analysis was carried out on a dual column—dual injector—dual detector system (Agilent 6890 N) with electron capture detection (ECD), and the injection was performed in pulsed splitless mode. The quantification was performed by external standard using a 6-point calibration curve (from 1 to 100 ng mL^−1^). Quality control procedures for HCB analyses included, in each batch of 20 samples processed, the analysis of a method blank and a spiked blank of two samples. In addition, certified reference material (NIST 1941B and NIST 1944) was analyzed for every five samples; further details are reported in Supplementary Table 1S.

### Chemical analyses in marine organisms

Mercury was determined in whole bodies of mussels and livers and muscles of different fish species. Samples were dried at 50°C overnight to constant weight and pulverized; approximately 0.5 g of homogeneous dried tissues were digested in a microwave digestion system (Mars-6 CEM, CEM Corporation, Matthews, NC, USA) using 5 mL nitric acid and 1 mL hydrogen peroxide. Mercury content was quantified by cold vapor atomic absorption spectrometry (Mercury Analyzer M-6100, Teledyne CETAC Technologies, Omaha, NE, USA). Quality assurance and quality control were assessed by processing blank samples, and reference standard materials (lyophilized mussel's tissue, Standard Reference Material NIST-2977, National Institute of Standards and Technology, Gaithersburg, MD, USA; lyophilized dogfish muscle, Standard Reference Material DORM-2, National Research Council, Ottawa, ON, Canada); further details are reported in Supplementary Table 2S. Concentrations obtained for standard reference materials were always within certified values' 95% confidence intervals.

For the determination of HCB, the organism samples were freeze-dried and homogenized, then an aliquot (2 g of tissues) was placed in an ASE 200 steel extraction cell, spiked with surrogate standards (CDN Isotope Inc. C13) for recovery monitoring, and extracted by pressurized fluid extraction system using a moderately polar solvent mixture. The resulting extract was cleaned up according to the matrix and the analytes by combining column chromatography on florisil^®^ adsorbents and shaking the extract with concentrated sulfuric acid. The cleaned-up concentrated extract was spiked with internal injection standard and analyzed by GC/MS/MS. Reference materials were analyzed to estimate the accuracy; further details are reported in Supplementary Table 2S.

### Biological parameters

#### Micronucleus test

The micronuclei (MNs) frequency was measured in the haemolymph of mussels *M. galloprovincialis* and gills of red mullet *M. barbatus* sampled in different sites from 2004 to 2013. After dissection, hemolymph from mussels and fish' gills were fixed in Carnoy's solution and maintained at +4°C until analyses; on the analysis day, separated cells were dispersed on glass slides and stained with the fluorescent dye 4',6-diamidino- 2-phenylindole at 100 ng mL^−1^. For each sampling site, hemolymph from 5 pools of mussels and gills from 6 to 8 fishes were investigated, and for each slide, 2000 cells with preserved cytoplasm were scored to assess the presence of MNs. MNs are round structures, smaller than 1/3 of the main nucleus diameter, on the same optical plan and separated from the nucleus (16, 36).

#### Cytochrome P450 induction (EROD activity)

The activity of 7-ethoxyresorufin O-deethylase (EROD) was measured according to ICES protocol (37) in the liver of red mullet *M. barbatus* collected in different sampling sites in 2003, 2004, 2008, and 2013. Livers were homogenized (1:5, w/v) in 100 mM K-phosphate buffer pH 7.5, 0.15 M KCl, 1 mM ethylenediaminetetraacetic acid (EDTA) and centrifugated at 12,000xg for 15 min, the resulting supernatants (S9) were immediately incubated at 30°C in a final volume of 1 ml containing 100 mM K-phosphate buffer pH 7.5, 4 μM 7-ethoxyresorufin, and 0.25 mM β-nicotinamide adenine dinucleotide (NADPH); 2 ml acetone were added after 5 min to stop the reaction. Incubation mixtures stopped at time zero were used as blank values and were subtracted from the sample fluorescence. Fluorimetric analyses (535/585 nm) were quantified by reference to resorufin standards (0.02–1 μM). Protein concentrations were measured according to the Lowry method, using bovine serum albumin (BSA) as standard. EROD activity is expressed as pmol/min/mg protein and mean values ± standard deviations.

### Statistical analysis

All statistical analyses were performed using RStudio (version 1.2.5033). Analysis of variance (2-way ANOVA) was applied to test differences between sites, sampling periods, and interactions “site × period” (level of significance at *p* < 0.05). One-way ANOVA was applied to biological parameters to test differences between sampling sites. Levels of significance were set at *p* < 0.05, homogeneity of variance was tested by Cochran C, and mathematical transformation was applied if necessary; *post hoc* comparison (Newman–Keuls) was used to discriminate between means of values. The results on micronucleus frequency were analyzed with the non-parametric Kruskal-Wallis test. Pearson correlation analyses were conducted on the whole dataset of chemical and biological parameters in *M. galloprovincialis* and *M. barbatus*.

## Results

### Sediments contamination

The concentrations of chemical contaminants in sediments (0–15 cm) over the years, including grain size, as percentages of gravel, sand, silt, and clay, are reported in Table 3, while the results of each core level are given in Supplementary material (SM1).

**Table 3:** Mean values of textural parameters and contaminants in superficial sediments (0–1 5 cm).

**Year**	**Area**	**Level (cm)**	**Gravel (%)**	**Sand (%)**	**Silt (%)**	**Clay (%)**	**Pelite (%)**	**Hg (μg g^−1^ d.w.)**	**HCB (ng g^−1^ d.w.)**
**EQS**								**0.3**	**0.4**
2003	S1	0–1 4	0.5	7.8	45.2	46.6	91.8	**8.3**	**34.4**
	S2	0–1 4	0.0	9.4	42.6	48.0	90.6	**28.0**	**150.0**
	S3	0–1 4	4.5	41.4	31.7	22.5	54.1	**13.1**	**222.4**
2004	S2	0–1 4	0.0	5.7	39.4	54.9	94.3	**55.7**	**209.5**
	S3	0–1 4	18.4	36.6	23.7	21.3	45.0	**83.0**	**447.3**
2008	S1	0–1 4	0.0	13.9	54.2	31.9	86.1	**4.5**	**13.4**
	S2	0–1 4	0.0	16.7	52.8	30.5	83.3	**79.0**	**2,525.6**
	S3	0–1 4	0.0	28.9	51.4	19.6	71.1	**82.9**	**4,493.1**
	S4	0–1 4	0.0	37.6	36.9	25.5	62.4	**11.4**	**158.2**
2013	S1	0–1 5	0.0	38.3	40.4	21.3	61.7	**4.1**	**26.9**
	S2	0–1 5	0.0	19.9	55.2	24.9	80.1	**114.2**	**1,186.5**
	S3	0–1 5	0.0	31.8	50.1	18.1	68.2	**92.7**	**1,412.0**
	S4	0–1 5	0.0	23.3	47.9	28.8	76.7	**13.4**	**103.9**

Environmental Quality Standards (EQS) for sediment according to Italian regulation D.lgs. 172/2015 are also reported.

In more coastal sites (S1, S2, S3), the average pelitic fraction was prevalent compared to the coarser ones (sand and gravel). The pelitic fraction (80.1–94.3%) prevails in almost all sampling years in S1 and S2, except for 2013, with a slightly lower value in the northernmost area (S1, pelite 61.7%); the sandy fraction was very variable over the years with percentages varying between 5.7% and 28.9%. A different trend was observed in the southernmost area (S3) during the first campaigns (2003 and 2004), with a coarse fraction accounting for >45.9%; in particular, in 2004, the gravel reached significant percentages (18.4%). In the outermost area (S4), the sediments' textural characteristics were very similar to those of the S3 area.

The Hg and HCB levels showed a clear spatial distribution with a positive gradient moving from the north (S1) to the south (S2–S3, Table 3). Concentrations were always high and widely exceeding (generally more than three orders of magnitude) the Environmental Quality Standard (EQS) defined under Directive 2000/60/EU as criteria for achieving the Good Chemical and Ecological Status of water bodies and established by the Italian regulation (Legislative Decree 172/2015; Table 3). In the northern area (S1), the concentrations of Hg and HCB were comparable between the different campaigns, ranging between 4.1 and 8.3 μg g^−1^ d.w. for Hg and between 13.4 and 34.4 ng g^−1^ d.w. for HCB. In the southernmost sector (S2 and S3), chemical concentrations increase, up to at least an order of magnitude, compared with those recorded in the northern sector. Hg concentrations increased from 2003 to 2013, with values ranging between 28 and 114.2 μg g^−1^ d.w. in the S2 area, while in S3, Hg concentrations were essentially constant from 2004 to 2013 (82.9-92.7 μg g^−1^ d.w.) and relatively lower in 2003 (13.1 μg g^−1^ d.w.) (Table 3).

The outermost area (S4) was characterized only in 2008 and 2013 with Hg levels of 11.4 and 13.4 μg g^−1^ d.w., respectively, lower than those measured in S2 and S3. High concentrations of HCB were also observed in all sampling sites in different years, with values exceeding two orders of magnitude the EQS (Table 3). In particular, the highest values were measured for HCB in 2008 and 2013 in S2 and S3, ranging from 1,186.5 to 4,493 ng g^−1^ d.w. The outermost areas (S4) and especially the northern one (S1) presented lower levels compared with the southern areas (S2–S3) (Table 3).

### Organisms' bioaccumulation

#### Mussels

Concentrations of Hg and HCB in native and transplanted mussels are reported in Table 4. Like for sediments, organisms showed a spatial distribution in contaminants levels, with lower values in organisms collected in the northern sector (1M) and higher in those sampled in the southernmost sectors (2–6 M, Table 4).

**Table 4:** Contaminant concentrations in native and transplanted mussels (*Mytilus galloprovincialis*).

***Mytilus***	**Year**	**Site**	**Hg**	**HCB**
***galloprovincialis***			**(μg g^−1^) d.w**.	**(ng g^−1^) d.w**.
**Reg. CE 1881/2006**		0.5	
Native	2003	CTRL	0.06 ± 0.007	0.7
		1M	2.03 ± 0.5	1.5
		2M	2.07 ± 0.06	6.3
		3M	5.01 ± 0.4	11.2
	2004	CTRL	0.05 ± 0.013	n.d.
		1M	0.61 ± 0.18	2.6
		2M	2.28 ± 1.21	11.2
		3M	2.72 ± 0.62	17.4
	2008	CTRL	0.015 ± 0.004	0.1
		1M	0.16 ± 0.019	0.7
		2M	1.23 ± 0.15	10.6
		3M	0.78 ± 0.16	12.0
		6M	1.07 ± 0.15	3.2
	2013	1M	1.19	0.7
Transplanted	2003	2T	3.01 ± 0.83	n.d.
	2004	2T	1 ± 0.01	3.6
		3T	0.75 ± 0.13	34.5
		4T	2.99 ± 0.52	10.9
		5T	0.87 ± 0.2	47.0
	2008	CTRL	0.015 ± 0.004	0.1
		1T	0.03 ± 0.05	0.6
		2T	0.05 ± 0.004	2.4
		3T	n.d.	n.d.
		6T	0.05 ± 0.012	1.4

The reference limit set by EU regulation for contaminants in seafood (CE 1881/2006) was also reported. Hg results are reported as mean ± standard deviation, n = 5. n.d., not detectable.

In particular, Hg was highly accumulated in native mussels (1M, 2M, 3M), especially in 2003, with values ranging between 2.03 and 5.01 μg g^−1^ d.w. Although Hg levels in mussels were still high, a slight temporal decrease was observed in native organisms collected in 2004 and 2008, with values almost halved as compared to 2003 (Table 3). Hg bioaccumulation in transplanted mussels also confirmed a different temporal bioavailability with higher values in 2003 than in the following sampling periods (Table 4).

A similar trend was also observed for HCB, with high values confirming clear bioaccumulation in mussels from all the sampling sites and within the period considered (Table 4). HCB concentration in native mussels confirmed spatial distribution within the bay, with higher values in organisms collected from the southern sector (3M). The environmental bioavailability of HCB was also highlighted by transplanted bivalves showing accumulation and high values, particularly in 2004 and lower in 2008, comparable to the trend observed in native organisms (Table 4).

#### Fish

To better compare the results obtained in different sampling years and considering the strong variability of fish species collected in different periods, the results showed in this manuscript only refer to red mullets, *Mullus barbatus* (Table 5), while those measured in other fish species are reported in Supplementary material (SM2). Hg concentration in the liver and muscle of organisms from the bay was usually higher than in controls and often exceeded the threshold limits set by EU regulation for contaminants in seafood (CE 1881/2006). The only exception is represented by *Boops salpa* and *Sparus aurata* collected in 2003 (1F) and *Boops salpa* sampled in 2004 (2F), which showed values in the muscle of 0.62, 0.95, and 0.18 μg g^−1^ d.w., respectively (SM2). Concerning *M. barbatus*, results on Hg bioaccumulation showed very high levels both in the liver and muscle of specimens collected in 2003, with values up to 26.09 and 8.62 μg g^−1^ d.w., respectively; clear bioaccumulation of Hg was still evident in *M. barbatus* collected inside the bay in 2004, 2008, and 2013 even if values were relatively lower than those measured in 2003 (Table 5). Elevated concentrations of Hg, without noteworthy variations among the sampling periods, were also observed for other fish species, with values ranging from 0.56 up to 31.42 μg g^−1^ d.w. in livers and from 0.18 to 7.99 μg g^−1^ d.w., in muscles (SM2). An elevated bioavailability was also highlighted for HCB, with some differences among the species (Table 5 and SM2). Tissue concentrations were particularly elevated in *M. barbatus* sampled in 2003 and 2004 and in *Diplodus* spp. sampled in 2004, with values ranging between 33.64 and 199.20 ng g^−1^ d.w. and between 8.70 and 70.70 ng g^−1^ d.w., respectively. HCB below EQS (10 ng g^−1^ w.w.) was measured in other fish species (SM2).

**Table 5:** Hg and HCB concentration in liver and muscle of *M. barbatus*.

***Mullus barbatus***	**Site**	**Year**	**Hg (μg g^−1^) d.w. liver**	**Hg (μg g^−1^) d.w. muscle**	**HCB (ng g^−1^) d.w. muscle**
**EQS**				**0.02**	**10**
**Reg. CE 1881/2006**				**1**	
	*CTRL*	2003	0.25 ± 0.1	0.37 ± 0.01	0.77
	*1F*		26.97 ± 9.68	8.62 ±2.59	33.64
	*1H*		9.75 ± 2.72	6.68 ± 1.77	191.1
	*CTRL*	2004	0.25 ± 0.1	0.37 ± 0.01	n.d.
	*1F*		5.83 ± 3.72	2.21 ± 0.22	199.2
	*3F*		3.45	2	6.8
	*2H*		8.46 ± 5.29	2.29 ± 0.88	46.1
	*CTRL*	2008	0.54 ± 0.18	0.14 ± 0.02	0.4
	*3H*		6.7 ± 1.4	2.32 ± 0.22	4.2
	*4H*	2013	12.09 ± 4.19	2.1 ± 1.2	6.78

The Environmental Quality Standard (EQS) for organisms and the reference limit set by EU regulation for contaminants in seafood (CE 1881/2006) were also reported. Hg results are reported as mean ± standard deviation. n.d., not detectable.

## Biomarker responses

**Figure 3:**
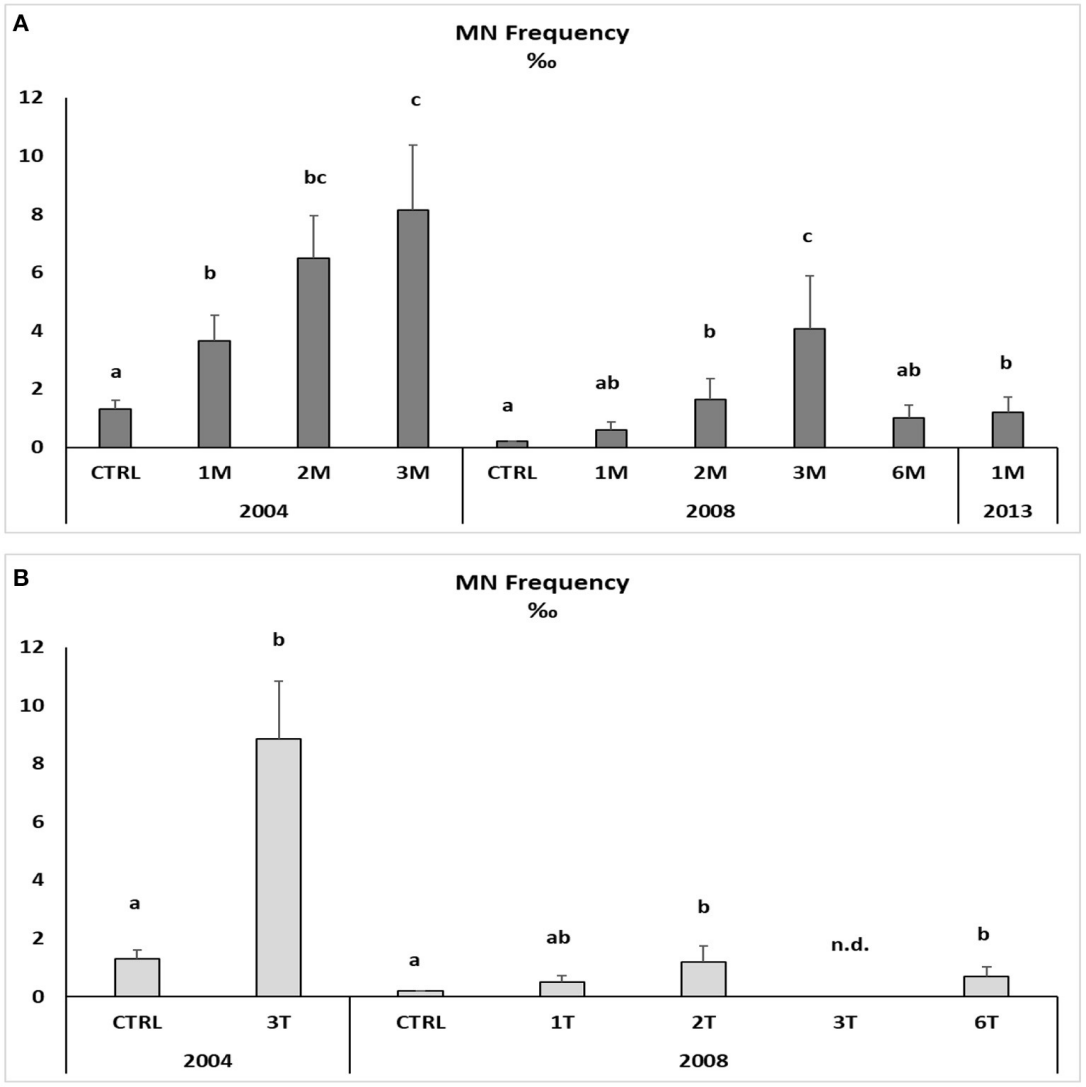
Micronuclei frequency in the haemolymph of native **(A)** and transplanted **(B)** mussels. Data are expressed as mean ± SEM and *n* = 5. Letters indicate differences among all the sampling sites for each year *p* < 0.01; n.d., not detectable.

Native and transplanted mussels collected in the bay revealed a significant increase in MNs frequency compared to control specimens, with remarkable differences among sampling sites and years (Figure 3; Table 6). The more evident effects were observed in 2004, with a MNs frequency ranging between 3.64 ± 0.9 and 8.85 ± 1.99 ‰ in native mussels (Figure 3) with higher values in organisms collected from the southern sector (3M) compared to those of the northern site (1M) (Figure 3). A temporal trend was also observed related to MNs frequency, with extremely high value in 2004 that was reduced in 2008 and 2013 even if in the last one mussels were collected only in the northern site (Figure 3). Transplanted organisms confirmed a high MNs frequency in organisms collected in 2004 and a strong frequency reduction of this nuclear anomaly in 2008, with values almost comparable to those observed in control mussels. In addition, significant correlations were observed for bioaccumulation of Hg and HCB and the onset of nuclear anomalies as MNs formation (SM3).

**Table 6:** The two-way analysis of variance results for chemical and biological parameters measured in mussels *M. galloprovincialis* and red mullet *M. barbatus*.

***M. galloprovincialis***	**Hg**	**MN**		
Site	***	***		
Period	***	***		
Site:period	***	***		
***M. barbatus***	**Hg liver**	**Hg muscle**	**EROD**	**MN**
Site	***	***	***	***
Period	***	***	n.s.	n.s.
Site:period	***	***	n.s.	n.s.

Asterisks indicate p < 0.001 and n.s., not significant.

**Figure 4:**
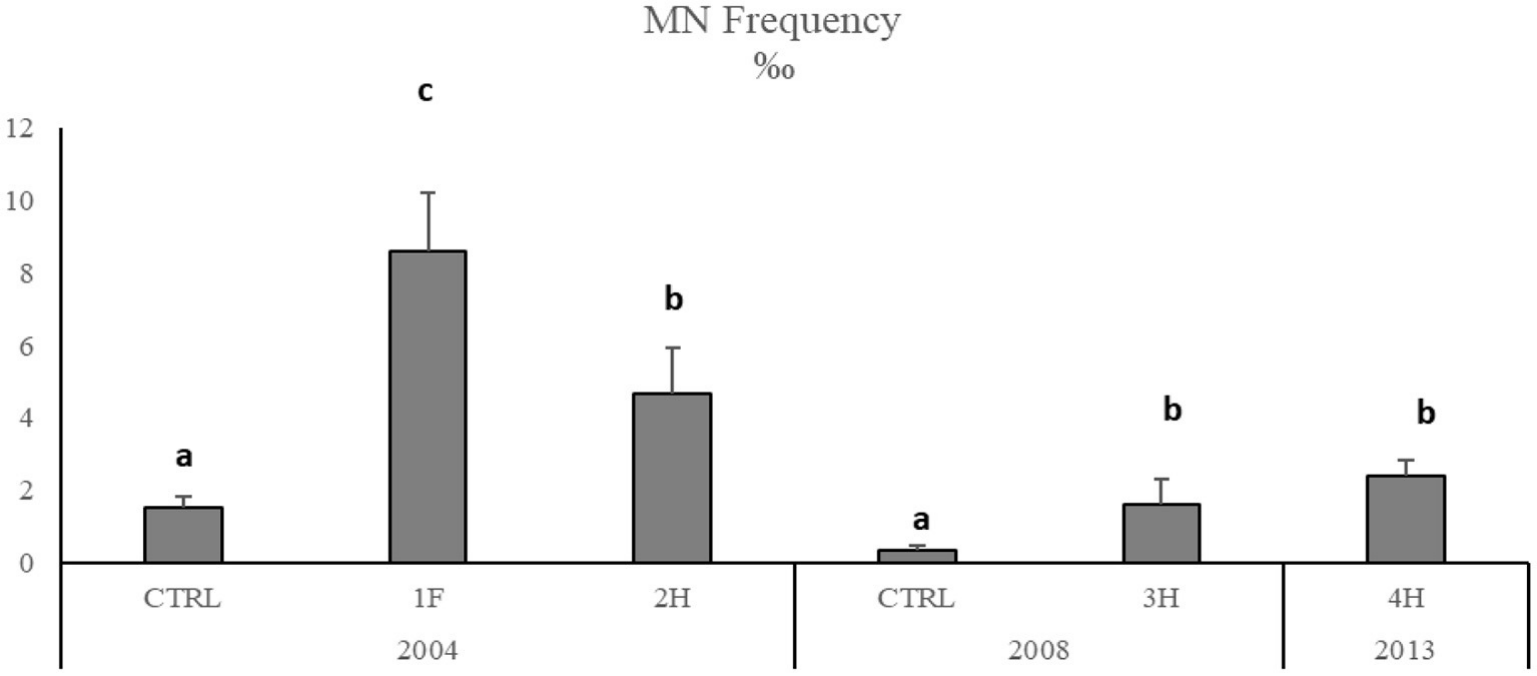
Micronuclei frequency in *M. barbatus* gills. Data were expressed as mean ± SEM and *n* = 5. Letters indicate differences among all the sampling sites, *p* < 0.01.

The onset of genotoxic effects was also measured in the fish's gills collected in 2004, 2008, and 2013 (Figure 4, SM2). As described for mussels and strictly correlated with Hg concentration in muscles (SM3), in *M. barbatus*, MNs frequency significantly increased within the Augusta Bay, with particularly high values in 2004, ranging between 1.6 ± 0.71 and 8.6 ± 1.62 ‰, and slighter, but still significant, in fish collected in 2008 and 2013 (Figure 4; Table 6). Results obtained for MNs frequency in other fish species showed values comparable to those measured in *M. barbatus*, generally ranging between 1.3 and 3.28 ‰ (SM2).

**Figure 5:**
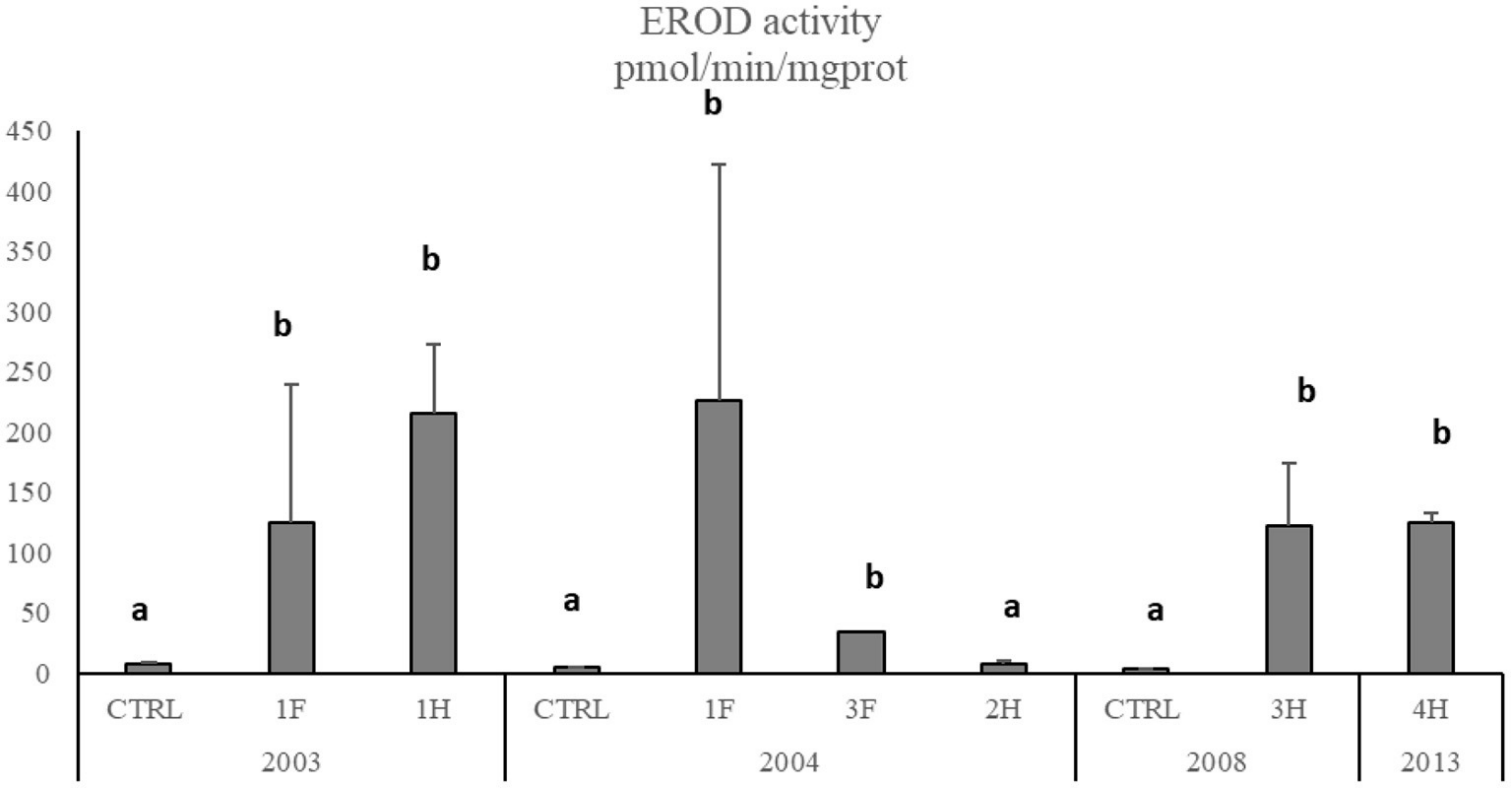
EROD activity in *M. barbatus*. Data are expressed as mean ± standard deviation. Letters indicate differences among all the sampling sites, *p* < 0.01.

The Cytochrome P450 showed a significant and strong increase of EROD activity in *M. barbatus* sampled in different sites, with values of one order of magnitude higher than those measured in control fish and ranging between 3.26 ± 0.27 and 226.7 ± 196 pmol/min/mg protein (Figure 5). No particular variations were observed among different years (Table 6), which always confirmed elevated responsiveness of biotransformation processes (Figure 5). Significant induction of EROD activity was also measured in the other fish species (SM2), with values particularly high in organisms collected in 2003 and 2004 and some specific spots in 2008 and 2013 for *Pagellus erythrinus* and *Sparus aurata* (SM2). The only exceptions were related to *Scorphaena* spp. and *Serranus* spp. lacking the induction of this biological system with values ranging between 2.65 and 7.57 pmol/min/mg prot (SM2).

## Discussion

In this study, a multidisciplinary approach integrating chemical analyses in sediments, bioaccumulation, and biomarker responses in target marine species was applied in a time window of 10 years to evaluate the environmental status, risk for marine organisms, and possibly for human health in one of the most polluted areas of the Mediterranean Sea. Chemical results on sediments confirmed those obtained in preliminary investigations highlighting that Augusta Bay is one of the most contaminated industrial sites, with high levels of Hg and HCB in sediments, by far exceeding the limits of Italian regulation (Legislative Decree 172/2015). Concentrations measured in the first 15 cm of sediment cores ranged between 4.1 and 114.2 μg g^−1^ d.w. and between 11.4 and 4,493.0 ng g^−1^ d.w. for Hg and HCB, respectively. In agreement with the previous studies, the chemical results on sediments confirm a gradient of contamination, from north to south, strongly influenced by the anthropogenic and industrial activities present in the area. As reported by ICRAM (28), sediments contained elevated concentrations of Hg (up to 198 mg kg^−1^ in the surface samples and up to 728 mg kg^−1^ in the deeper layers) and HCB (up to 5 mg kg^−1^) in the southern part of the bay, close to the industrial pole and chlor-alkali plant. Similar results were obtained by Romano et al. (38, 39), showing significant contamination in superficial sediments of the southern area due to Hg, PAHs, and PCBs (up to 322, 14.60, and 3.75 mg kg^−1^, respectively), as further confirmed by other studies (2, 27, 40–43). The use of radiometric methods (137Cs) allowed to reconstruct the contamination chronology and demonstrate that the exceptionally high concentrations of Hg and HCB were attributable to the historical activity of a chlor-alkali plant while PCBs were likely derived from petrochemical plants (27). This contamination affected the entire bay to varying degrees, with a contamination transfer based on concentrations related to depths and decreasing in the sediment cores from the southernmost sector to the central and northern areas (29). No temporal trend nor significant variations were observed for levels of these chemicals in sediments. The persistence of such extremely high concentrations of contaminants in surface sediments of the southern area after the closure of the chlor-alkali plant in 2003 represents an environmental health concern suggesting some degree of sediment redistribution or resuspension possibly caused by shipping activities. The elevated concentration of Hg, potentially transformed into methyl-mercury (MeHg), and HCB can accumulate in marine organisms and biomagnify into the food web *via* benthic-pelagic coupling (7, 44). The possible trophic transfer of chemicals from sediments to biota and the bioavailability of investigated pollutants (Hg and HCB) were evaluated by measuring their accumulation in several bioindicator organisms, such as native and caged mussels *M. galloprovincialis*, red mullets *M. barbatus*, and other local fish species. The results from native mussels showed elevated levels of Hg and HCB in organisms, confirming a gradient with a higher bioaccumulation rate in the southern area than in the northern one. In addition, a general temporal decrease of Hg and HCB accumulation was observed after 2004, highlighting a gradual reduction of chemicals availability after the closure of the chlor-alkali plant in 2003.

Results on transplanted mussels confirmed the temporal trend in the bioavailability of Hg and HCB. The concentrations of these chemicals accumulated in 2003 and 2004, with values comparable to those of native mussels, were significantly lowered in 2008, with values slightly above the controls. Red mullets confirmed a marked accumulation of Hg and HCB and, despite being less evident than in mussels, a certain decrease in chemical availability after 2004. These data support the hypothesis that the reduction of contaminant bioavailability was greater in the water column than in the sediments, as confirmed by previous studies showing that the flux of Hg at the sediment-water interface depends on the speciation of chemical compounds in sediment to guarantee QA/QC thus acting as a Hg source for the overlying water column and, consequently, for the whole Mediterranean Sea (30, 31). Concentrations of Hg in *M. barbatus* were much higher in livers than in muscles, confirming a strong sediment-related bioavailability of this element during the investigated time window (45). Liver concentrations typically depend on the current bioavailability of chemicals, while Hg in muscle reflects the long-term accumulation of this element influenced by the affinity of methyl-Hg for lipids of muscle tissues (46). Concerning organic compounds, the HCB concentrations measured in organisms were comparable to those measured in other polluted sites, i.e., Taranto (47), different areas of the Iberian Peninsula, Mediterranean Sea (48), Ebro Delta (Western Mediterranean), Gulf of Naples, Southern Tyrrhenian Sea and Samsun region and Gulf of Izmir, Turkey (49).

Despite the gradual temporal decreases in bioavailability, Hg concentrations measured in mussels and especially in *M. barbatus* exceed both the reference limit set by EU regulation for contaminants in seafood (CE 1881/2006) of 0.5 and 1 mg kg^−1^, respectively, with the only exception of caged mussels collected in 2008 and fish collected from 2F site in 2004. Furthermore, Traina et al. (2) confirmed that Hg concentrations in fish were particularly high in 2017, suggesting a potential risk for human health associated with food consumption, despite fishing activities being officially banned in this area.

On the other hand, Ausili et al. (17) had already focused on the real and concrete risk to human health from the consumption of contaminated fish species in the Augusta area: International Agencies indicate provisional tolerable weekly intake (PTWI) of Hg, ranging from 0.7 μg kg^−1^ body weight (b.w.) (50) to 1.6 μg kg^−1^ b.w. (51). These limits represent safe values that the human population can generally ingest over a lifetime, even though different considerations are recommended for pregnant women, nursing mothers, and young children; due to the toxic effects of Hg on the developing nervous system, these categories should avoid the consumption of fish species which normally have higher mercury concentrations (52, 53). In this respect, Ausili et al. (17) estimated a median Hg value for fish muscle (various species) from the Augusta area ranging from 1.43 μg g^−1^ (w.w.) when considering all the sites to 2.06 μg g^−1^ (w.w.) for only the most impacted. Consequently, a 60 kg woman would assume a Hg PTWI (0.7–1.6 μg kg^−1^) eating from 20 to 67 g of fish caught in different sites of the industrial area of Priolo. Since the national average of fish consumption for the Italian population is estimated to be 33.9 g per capita per day [~240 g per week, (54)], a 60 kg women consuming organisms from the Augusta-Priolo area would exceed the Hg PTWI by at least 4 to 12-fold (17). In addition, criteria developed to protect human health from mercury in fish products indicate that calculations for pregnant women should consider a fish consumption 3-fold higher than the national average (50). Applying this cautionary procedure would further exceed the recommended safe limits for the area of Priolo, confirming consumption of seafood as a serious concern for pregnant women (17). The hypothesis of human health risk was supported by the frequency of neonatal malformations, which in 2001 was 5.5% for the area of Augusta-Priolo versus a national average of 1.5% and a risk value indicated by WHO at 2% (55).

The importance of an ecotoxicological approach and early and sensitive biomarkers is widely recognized for monitoring the adverse effects caused by acute and chronic pollution (15, 16, 18, 56). The cytochrome P450 is of central importance to the metabolisms of many xenobiotics, and the induction of EROD activity is considered a specific biomarker of fish exposure to organic pollutants, including HCB (57). The biotransformation of environmental contaminants significantly affects their impact on organisms' health. Overall, results showed a strong enhancement of EROD activity in *M. barbatus* in 2003, with values 100–200 times higher than those measured in control organisms, indicating a clear induction of the biotransformation pathway, as also observed in other fish species. The CYP induction was confirmed in 2004 with values comparable to 2003, while in 2008 and 2013, EROD induction was still significant but lesser: these results indicate a lower but permanent bioavailability of organic compounds, even 10 years after the closure of the chlor-alkali plant. The EROD activity measured in this study in *M. barbatus* was comparable to that observed in *Zosterisessor ophiocephalus* collected from the Venice Lagoon (58, 59), Bizerte Lagoon, Tunisia (57), and in *M. barbatus* collected from a polluted site of Otranto-Adriatic coast (60), in a sediment dumping site in the Tyrrhenian Sea (13), and organisms exposed in laboratory conditions to organic compounds and elutriate obtained from Porto Marghera (Venice Lagoon) sediments (61, 62).

The induction of Cytochrome P450 caused by organic compounds and the presence of prooxidant trace metals such as Hg could unbalance the oxidative status of marine organisms (62, 63) and consequently affect biological macromolecules as lipid, proteins, and DNA (64). Oxidative stress has been related to DNA strand breakages and MNs induction in mussels and fish (48, 65–68). The application of the MN test in mussels has been recommended as a nonspecific biomarker of genotoxicity in marine pollution assessment (69). Our results showed a general increase of micronuclei frequency both in native and transplanted mussels and in *M. barbatus* collected in 2004 when the bioavailability of Hg and organic compounds was the highest, immediately after the closure of the chlor-alkali plant. In particular, the MNs frequency in mussels presented the same geographical trend observed for chemical bioavailability, suggesting a strong correlation between these parameters. The correlation between xenobiotics bioavailability and genotoxic damage is also confirmed by the induction in MNs formation in caged mussels with values comparable to those measured in native organisms. A general decrease in MNs formation was observed in mussels from 2008, paralleling the concomitant reduction in contaminants bioavailability, especially Hg. A similar temporal trend was also observed for *M. barbatus* that in 2013 exhibited an MNs frequency comparable to that of control fish.

## Conclusions

In this study, the integrated approach was demonstrated to be an effective tool for assessing the environmental persistence and the risk for marine organisms and human health in a heavily polluted marine area. The analyses of several parameters over a wide time window provided the chance to observe the temporal trend of the legacy pollution in the different matrices through the adverse effects on the marine organisms.

These results may help better understand the evolution of other marine coastal areas affected by a similar history of contamination and suggest potential environmental remediation and mitigation actions.

## Data availability statement

The original contributions presented in the study are included in the article/Supplementary material, further inquiries can be directed to the corresponding author/s.

## Author contributions

Led conception, writing and design of the manuscript, and data analysis and interpretation: AA, MB, and ER. Sampling activity: AA, MB, DF, SG, FR, and ER. Sediment analysis: CM, DSM—Hg, and GS—HCB. Organisms' analysis: DF—Hg and GS—HCB. Biological parameters: MB and SG. Images drawing: AS. All authors listed have made a substantial, direct, and intellectual contribution to the work and approved it for publication.

## Conflict of interest

The authors declare that the research was conducted in the absence of any commercial or financial relationships that could be construed as a potential conflict of interest. The handling editor FB is currently organizing a research topic with the author MS.

## Publisher's note

All claims expressed in this article are solely those of the authors and do not necessarily represent those of their affiliated organizations, or those of the publisher, the editors and the reviewers. Any product that may be evaluated in this article, or claim that may be made by its manufacturer, is not guaranteed or endorsed by the publisher.
