# Corrigendum: 10-year time course of Hg and organic compounds in Augusta Bay: Bioavailability and biological effects in marine organisms

**DOI:** 10.3389/fpubh.2022.1080886

**Published:** 2022-11-22

**Authors:** Maura Benedetti, Elena Romano, Antonella Ausili, Daniele Fattorini, Stefania Gorbi, Chiara Maggi, Andrea Salmeri, Daniela Salvagio Manta, Giulio Sesta, Mario Sprovieri, Francesco Regoli

**Affiliations:** ^1^Department of Life and Environmental Sciences, Polytechnic University of Marche, Ancona, Italy; ^2^CoNISMa, Consorzio Interuniversitario per le Scienze del Mare, Rome, Italy; ^3^ISPRA, Italian Institute for Environmental Protection and Research, Rome, Italy; ^4^Institute of Anthropic Impacts and Sustainability in Marine Environment, National Research Council, Trapani, Italy

**Keywords:** mercury, hexachlorobenzene, marine organisms, trophic transfer, biomarkers, bioavailability

In the original article, Supplementary material [Supplementary material 1, Supplementary material 2] were mistakenly not included in the publication. The missing materials have been published.

[Supplementary material 1, Supplementary material 2]

The authors apologize for this error and state that this does not change the scientific conclusions of the article in any way. The original article has been updated.

## Publisher's note

All claims expressed in this article are solely those of the authors and do not necessarily represent those of their affiliated organizations, or those of the publisher, the editors and the reviewers. Any product that may be evaluated in this article, or claim that may be made by its manufacturer, is not guaranteed or endorsed by the publisher.

